# Transcriptional profiling of the *M. complexus* in naked neck chickens suggest a direct pleiotropic effect of *GDF7* on feathering and reduced hatchability

**DOI:** 10.1186/s12864-024-10965-0

**Published:** 2024-11-15

**Authors:** Alexander Charles Mott, Carina Blaschka, Andrea Mott, Clemens Falker-Gieske

**Affiliations:** Department of Animal Sciences, Georg-August-University Göttingen, Göttingen, Germany

**Keywords:** Hatchability, RNASeq, GDF7, PITX2, Transcription factors

## Abstract

**Background:**

The locus for naked neck (*Na*) in chickens reduces feather coverage and leads to increased heat dissipation from the body surface resulting in better adaptability to hot conditions. However, the *Na* gene is linked to significantly lower hatchability due to an increased late embryonic mortality. It has been argued that the causative gene *GDF7* may have a direct pleiotropic effect on hatchability via its effect on muscle development. Thus, the study presented here analyses the transcriptome of the hatching muscle (*M. complexus)* and shows how *GDF7* impacts development leading to reduced hatching rates in *Na* chickens.

**Results:**

Using 12 chicken embryos (6 x wildtype (*Wt*) and 6 x *Na*) RNA was extracted from the *M. complexus* of each embryo and sequenced. The resulting differential expression analyses led to the discovery of 461 differentially expressed (DE) genes in the *M. complexus* of the experimental group. Among those, 77 genes were of uncertain function (LOC symbols), with 31 were classified as uncharacterised. The regulation of a number of pathways involved in normal embryonic development, were found to be negatively influenced by the *Na* genotype. Further pathways involved in cell-cell adhesion, cell signalling pathways, and amino acid (AA) metabolism/transport were also observed. *GDF7* (alias *BMP12*), whose localised overexpression in the neck skin causes the *Na/Na* phenotype, was significantly overexpressed in the *M. complexus of Na/Na* embryos, and shows a significant increase in the number of binding sites for the transcription factor *PITX2* was also observed.

**Conclusion:**

In *Na* chickens, *GDF7* is under the control of a mutated cis-regulatory element, whose actions are known to suppress the development and distribution of feathers through the sensitizing action of retinoic acid. In this study, a number of DE genes with over 10 retinoic acid response elements (RAREs) in close proximity were observed, indicating changes to the retinol metabolism. With the understanding that the *Na/Na* mutation leads to increased retinoic acid activity, this indicates a high likelihood of *GDF7* excerpting a direct pleiotropic effect, not just in the observed reduction in feather patterning, but also impacting the development of the *M.complexus*, and consequently leading to the reduced hatchability observed in birds with the *Na/Na* genotype.

Furthermore, the enrichment of *PITX2* binding sites in proximity to DE genes in the *M. complexus,* also indicates that muscle development is still ongoing in *Na* embryos. This suggests that the *M. complexus* is not yet fully developed, further increasing the potential for late embryonic mortality in *Na* chicks at hatching.

**Supplementary Information:**

The online version contains supplementary material available at 10.1186/s12864-024-10965-0.

## Background

With the expected rise in global temperatures, and extreme heat events occurring in ever increasing numbers, this presents growing challenges for poultry and egg producers [1–3]. As temperatures become elevated, current production animals are poorly equipped to deal with the increased stresses presented, negatively effecting functional traits such as feed consumption and efficiency levels, as well as metabolic changes leading to reduced carcass and egg weights, lower egg production numbers, and altered protein levels in chickens [4–11]. Physical steps such as cooling regimes and increased animal spacing, can be implemented for “indoor” birds, but with increased production energy usage and added costs to the producer [12–14]. In addition, the current production trends in consumers is moving away from intensive indoor farming, towards a perceived return to outdoor, smaller ”free range” poultry farming [15]. The impact of this would mean animals being exposed to increased temperatures without the possibility of external temperature control. As stated, this not only effects adult birds, but has a visible effect observed in egg production, fertility and hatchability [16–18]. Interestingly, some naked neck broiler breeds have shown an increased ability to cope with increases in temperatures, due to their reduced feather coverages and comparatively lower metabolic performance compared to commercial breeds [6, 17, 19]. Although in comparisons between commercial and naked neck cross breeds in a heat stress environment, an increased laying rate, egg quality and rate of fertilisation have been observed [20–22]. A significant reduction in hatchability and increase in late embryonic mortality has also been witnessed in naked neck breeds at normal temperatures [17, 23]. The potential, therefore, of introgressing the naked neck allele into a commercial strain has the possibility to increase heat tolerance and have beneficial effects on growth rates in commercial broiler strains, but also has the potential to compromise the breeding of such hens.

Hatching starts around days 19 and 20, the embryo partakes in internal pipping, followed by external pipping on day 21. In order for this to be successful, the chick needs to use the egg tooth and hatching muscle (*M. complexus*) to open a spot of the eggshell from which it can emerge [24, 25]. Here, the hatching muscle acts as a cushion for the head and by contracting elevates the beak, holding it in one place, aiding in the pipping process [26, 27]. From day 15 of incubation the *M. complexus* increases in size, reaching its maximum shortly before hatching on day 18/19 of incubation, and swiftly decreasing during the initial days after hatching [28]. Whilst the causal mutation of the *Na* variant has been shown to be a large insertion of ~180bp from Chromosome 1 at ~260kb downstream of a protein coding gene for *GDF7* [29], Mou *et al.* (2011) further identified elevated levels of the bone morphogenetic protein *BMP12* (also known as *GDF7*) to be responsible [30]. Although bone morphogenetic proteins (BMPs) play a general role in the embryonic development [31], this does not give a clear explanation for the lower hatchability associated with *Na/Na* chickens. With the essential role attributed to the *M. complexus* in the hatching process, the question of the impact of the *Na* genotype on this tissue still remains unanswered [17]. Moreover, the BMP family of genes have been shown to have a pleiotropic effect in a number of different tissues and organisms [32, 33], not yet seen with GDF7 in chickens. As such, this study analyses the transcriptome of the *M. complexus* in *Na/Na* chicken embryos and aims to show how the pleiotropic effect of GDF7 can lead to restricted development and the reduction in hatching rates observed in *Na/Na* chickens. Samples from chickens derived from a heterozygous parental generation were used to perform a comparative transcriptomic analysis. This study indicates changes in gene expression in the *M.complexus* are due to the *Na/Na* genotype and elucidates whether *GDF7* has a pleiotropic effect in the *M.complexus* and how that can impact hatchability.

## Methods

### Ethical statement

No animals were sacrificed and anaesthetised in this study, which was carried out in accordance with relevant national and international guidelines, under current ethical clearance (file number E10-19), and with good veterinary practice applied to all handling procedures.

### Animal husbandry

A parental generation of six cocks and eight hens with heterozygous *Na/Na* genotype were utilised as the breeding base for this study.

Animals of the parental population were derived from a local chicken breeder and were hatched and reared at the Department of Animal Science, Georg-August-University Göttingen. Birds were held in groups separated by sex in environmentally controlled aviaries with 19.1 ± 0.3°C room temperature. Each aviary contained round feeding troughs and drinkers, and the floor was covered with a layer of straw.

During the experimental period, a rotational mating system was implemented in order to reduce the possibility of any sire effect. This involved rotating the cocks every 24hr. The eggs were then collected twice a day over nine consecutive days. This was then repeated on five separate occasions. The eggs were then stored at 17.3 ± 0.2°C until incubation was initiated.

In the afternoon of collection day nine, egg incubation was started. Brooding took place for 20 days in an incubator (Euro-Lux Paris, HEKA-Brutgeraete, Rietberg Germany) at 37.8 °C and 55% air humidity from day one to 18 and 65% air humidity up to the sample collection. Six to seven days after start of brooding and before the sample collection eggs were screened for fertilisation through candling. For each replicate, the fertilisation rate was calculated.

### Sample collection

Muscle samples (*M. complexus / M. pectoralis superficialis*) were obtained from individual embryos at day 20 of embryonic development before hatching. The shell of each egg was opened at the blunt pole, the membrane was opened and the embryo was removed from the shell. Afterwards, the embryos were immediately decapitated and the skin of the neck and breast region was removed and the muscle exposed. A piece was collected from the liver and the hatching muscle/*M. complexus* for RNA extraction, and stored in RNAlater (Invitrogen, ThermoFisher Scientific, Osterode am Harz, Germany). *M. pectoralis* tissue was collected for DNA extraction and stored at -20°C.

### Genotyping and sexing

Genotyping for *Na* and sexing were carried out as previously outlined [30, 34].

DNA was extracted from the *M. pectoralis* using the DNeasy Blood and Tissue Kit (Qiagen, Hilden, Germany) according to the manufacturer’s protocol and immediately used for following PCR. The DNA concentration was determined employing the Infinite M200 Pro (Tecan, Crailsheim, Germany).

The presence or absence of the target region in each animal was determined using polymerase chain reaction analysis (PCR), based on Mou *et al* (2011) for genotyping, and He *et al* (2019) for sexing [30, 34]. The reaction mix contained 10x PCR buffer, 50 mM MgCl_2_, 10mM dNTP mix, 10µM of each primer, 50ng DNA template, Taq polymerase (ThermoFisher Scientific, Osterode am Harz, Germany), with water added to achieve a final volume of 25µl. The PCR program employed an initial step of 94°C for 3min followed by 35 cycles of 45sec at 94°C for denaturation, 30sec at different temperatures (Supplementary Table S1) for annealing of primers, and extension at 72°C for 1-3 min depending on the primer, each. The last cycle was followed by a 10min final extension at 72°C and cooling to 4°C. Finally, PCR products were subjected to electrophoresis on a 1% agarose gel in 1x TBE buffer containing 6µl Green stay (HD Green, Intas Science Imaging Instruments, Göttingen, Germany). The gel image was visualised and recorded using Intas Science Imaging system (Intas Science Imaging Instruments, Göttingen, Germany) (Supplementary file S2.).

### RNA extraction

For RNA isolation 900μl Qiazol (Qiagen, Hilden, Germany), 5μl DX reagent and 20–30 ceramic beads were added to 10–30mg of collected tissue. The mixture was placed in a Bead Ruptor (Omni-International, Kennesaw, Georgia, USA) and homogenized with the following program: 4.5m/sec, 3 times 15sec, 10sec dwell. After 5min incubation at room temperature, 100μl gDNA Eliminator solution (Qiagen, Hilden, Germany) was added and the sample was vortexed for 15sec. After addition of 180μl chloroform the sample was vortexed for 15sec and incubated at room temperature for 3min. Samples were centrifuged at 12,000g and 4°C for 20min and aqueous phase was transferred to a fresh reaction tube. An equal amount of 70% ethanol was added to each sample and vortexed for 15sec. RNA was purified using the RNeasy Plus Universal Mini Kit (Qiagen, Hilden, Germany) according to the manufacturer’s protocol and stored at − 80°C.

### RNA sequencing

Input total RNA was visualized on a TapeStation 4200 (Agilent, Waldbronn, Germany) and all but three samples respectively showed excellent RNA quality with RIN-scores > 8 (RIN scores of 6.8, 6.9 and 7.7). Samples were quantified on a Qubit 2.0 Fluorometer (ThermoFisher Scientific, Osterode am Harz, Germany) and 500ng of total RNA was used as input for the TruSeq stranded mRNA library kit (Illumina, Berlin, Germany) following the manufacturers manual. Resulting libraries showed a fragment size distribution of around 300bp and were sequenced 2 × 75bp paired-end. Sequencing was conducted on the HiSeq 4000 (Illumina, Berlin, Germany) with 10 samples / lane.

### Differential gene expression analysis

Differential gene expression analysis was performed using the homozygous *Wt* genotype as the baseline dataset for comparing the expressed genes of the *Na/Na* genotype. Raw sequencing reads were processed with Trimmomatic version 0.36 [35] for adapter removal, trimming of low-quality base calls, and removal of low-quality reads. Using the following settings: PE -phred33 LEADING:3 TRAILING:3 SLIDINGWINDOW:4:15 MINLEN:36. Read pairs were discarded if one read did not survive quality control. Trimmed reads were aligned to chicken genome version GCF_016699485.2, using Hisat2 version 2.1.0 [36] with default settings. Splice sites were derived from the gene transfer format (GTF) file. FeatureCounts of the Subread package (Version 2.0.0) [37] was used to count exon spanning reads. Differential expression analyses were conducted with DESeq2 (Version 1.40.1) [38]. Volcano plots of differential expression analyses were created with the R package EnhancedVolcano (Version 1.6.0) [39]. All R packages were obtained with Bioconductor version 3.11 [40].

### Functional analyses

Gene clusters were compared and visualised with the R package clusterProfiler [41]. Gene symbols were translated into ensemble IDs through the clusterProfiler Biological Id Translator (bitr). Gene ontology (GO) term analyses were conducted using enrichGO (settings: pAdjustMethod = "fdr", pvalueCutoff = 1, qvalueCutoff = 0.25, readable = TRUE, minGSSize = 10). KEGG pathway analysis was performed using enrichKEGG (settings: pvalueCutoff = 1, pAdjustMethod = "BH", minGSSize = 10, maxGSSize = 500, qvalueCutoff = 0.25, use_internal_data = FALSE) and plots were generated using the dotplot function. Protein interaction maps were constructed with STRING (Version 11.0) [42] employing default settings.

### Retinoic acid response element (RARE) analysis

The discovery of retinoic acid response elements (RAREs) was described in our previous study [43]. Briefly, RAREs within 10kb upstream of transcript start and 10kb downstream of transcript end were counted for each gene annotated in the chicken reference genome. This list of RAREs was then compared to the significant DE genes found in the *M. complexus* of *Na/Na* embryos (Supplementary Table S3).

### Transcription factor enrichment analysis

Transcription factor enrichment analysis was performed with CiiiDER (build May 15th 2020) [44]. DE genes (Muscle = p-adj. < 0.01, abs. Log_2_FC > 1) were used as input for the screening of transcription factor binding sites. All the transcripts with an abs Log_2_FC < 0.2 were used as background genes. The *p*-value threshold for gene coverage enrichment was set to 0.05, base position upstream scan limit to 1500bp, and base position downstream scan limit to 500bp. To predict transcription factor binding to a target DNA sequence, a position frequency matrix (PFM) is required, which assigns nucleotide frequencies to each position in the binding motif. All transcription factors that were DE in muscle were screened for binding site enrichment in proximity to the DE genes. All PFMs were acquired in JASPAR format [45] choosing the first *Homo sapiens* profile available, and a comprehensive list can be found in Supplementary Table S4. The transcription factor enrichment plot was created within CiiiDER [44].

## Results

### Genotype and sexing parameters

The embryos collected showed a slight shift towards the *Na/Na* genotype. From a total of 87 embryos collected from 5 independent rounds of mating 19 embryos (21.84%) were found to be *Wt*, 29 (33.33%) *Na/Na* and 39 (44.83%) heterozygous (*Wt/Na*), with an overall average split of almost 50%, 49.43% female and 50.57% male. Using the collected parameters, 12 selected chicken embryos (3 *Na/Na*-male, 3 *Na/Na*-Female, 3 *Wt*-Male, 3 *Wt*-Female) were randomly selected and RNA from the liver and *M.complexus* was analysed. These two genotypes were chosen to give the best chance to observe transcriptional changes affecting the *Na/Na* embryos.

### Differential expression analysis of *Na/Na* and *Wt* chickens

**Fig. 1:**
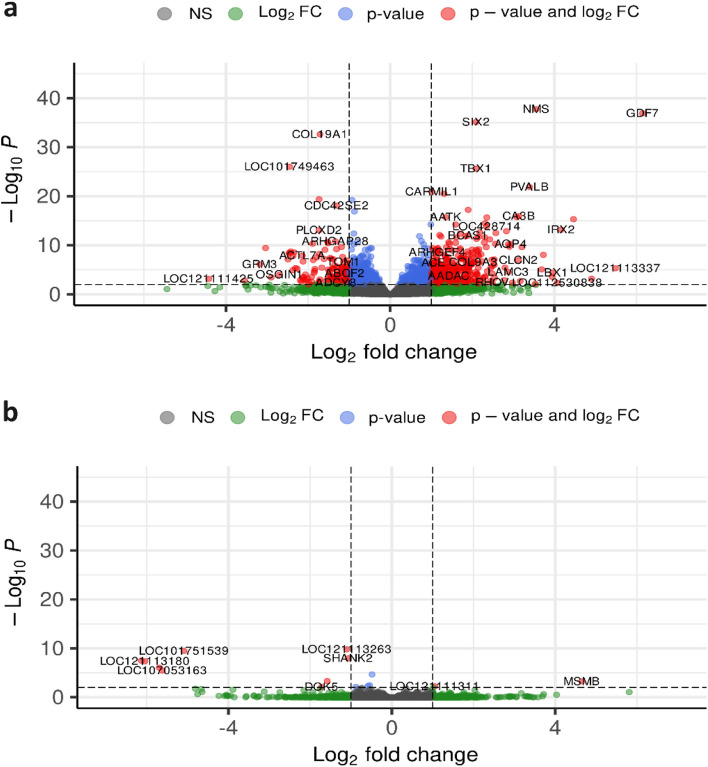
Volcano plots of genes differentially expressed in *M.complexus* (**a**) and Liver (**b**) samples from naked neck chicken embryos compared to wild type embryos. Grey dots represent transcripts that were not differentially expressed, green transcripts were above the absolute log_2_ fold change threshold of 1, blue transcripts were below an adjusted *p*-value of 0.01, and red transcripts were above the threshold of 1 and below the adjusted *p*-value of 0.01. Log2 fold change and adjusted *p*-value thresholds are indicated by the dashed lines

This analysis indicated that there was a total of 25,081 genes identified from the 12 analysed embryos (average FPKM > 1). Of these 25,081 genes expressed in the samples, 461 were DE in the *M. complexus* (abs. Log_2_FC ≥1, padj < 0.01). 338 of the DE genes were found to be up regulated in the *Na/Na* chickens and 123 were down regulated, (Fig. 1a). Among those, 77 genes of uncertain function (LOC genes) were found (52 upregulated / 25 downregulated), with 46 LOC genes having possible functions, and 31 being classified as non-coding RNAs (ncRNAs) or uncharacterised. Comparatively, the liver samples had only 11 DE genes observed to be different in *Na/Na* embryos, 8 of which were LOC genes, and all were classified as ncRNAs (Fig. 1b).

### Functional pathway and protein interaction analysis of differentially expressed genes in the *M. complexus of Na/Na* embryos

**Fig. 2:**
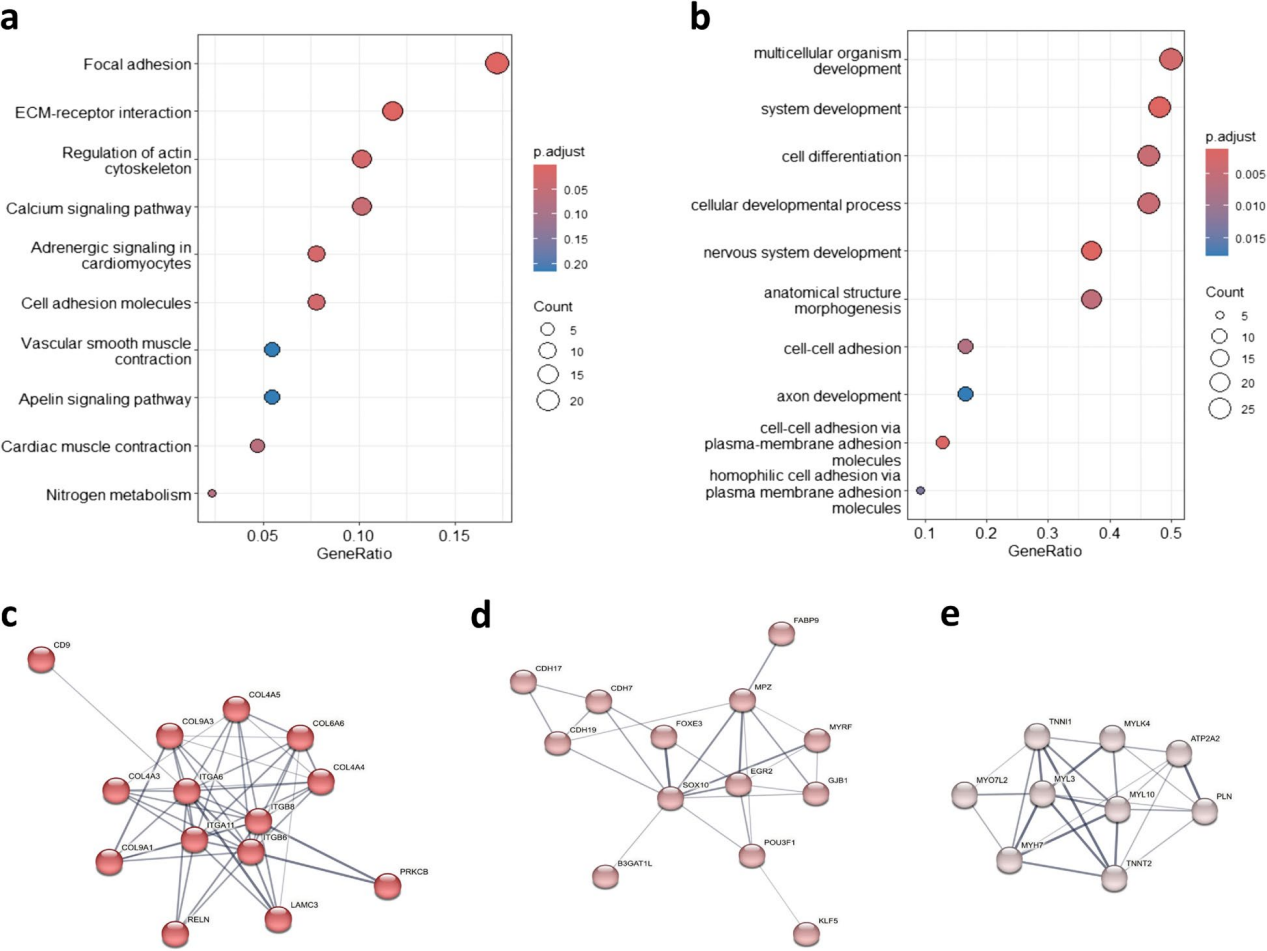
Gene cluster analysis results for genes differentially expressed in *M.complexus* of naked neck embryos compared to wild type embryos for KEGG pathways (**a**) and biological processes (**b**). Protein enrichment analysis of differentially expressed gene clusters for collagen formation (**c**), cell-cell adhesion (**d**), and muscle cell contraction (**e**)

DE genes between *Na/Na* and *Wt* embryos were analysed, and a number of KEGG pathways and cellular component-based GO terms were found. The enlightened KEGG pathways were related to focal and cellular adhesion, cell signalling pathways, muscle contraction, and nitrogen metabolism (Fig. 2a). The observed biological processes were linked to developmental processes, cellular adhesion, and cell differentiation (Fig. 2b). Furthermore, the results from the protein interaction analysis gives further weight to the previous results clearly showing the presence of 3 large protein interaction clusters relating to collagen formation (Fig. 2c), cell-cell adhesion (Fig. 2d), and muscle cell contraction (Fig. 2e). Smaller clusters of 4 and 5 genes were also found covering AA metabolism and transport, signalling pathways, focal adhesion, and transmembrane transport were also observed (Supplementary Table S5a-m).

### Gene regulation through retinoic acid suppression

Comparing DE genes to a previously compiled list of RAREs, highlighted 240 genes that had RAREs within 10kb upstream of transcript start and 10kb downstream of transcript end (Supplementary Table S6). RAREs were found close to the coding region of 15 of the 240 observed DE genes, with *LSAMP* and *LOC107051325* having more than 20 (Table 1). The average occurrence of RAREs is 0.77 per gene [43].

**Table 1:** Differentially expressed genes found in close proximity to RAREs at an occurrence of 10 or more per gene, including gene symbol, occurrence count, log_2_ fold change (LFC), and differential significance (*p*-value) for the *M.complexus* of naked neck embryos compared to wild type embryos

Symbol	Count	LFC	*p*-value
*LSAMP*	28	1.310028	1.57E-24
*LOC107051325*	25	1.97174	6.93E-09
*CACNA1C*	16	1.336966	1.54E-06
*KLHL29*	16	-1.06557	1.02E-09
*MDGA2*	15	1.35298	5.47E-06
*LOC112531762*	14	-2.12848	4.41E-05
*GRID1*	13	-1.12611	0.000362
*RELN*	13	1.130771	0.000191
*SORCS1*	13	1.207938	0.000141
*SLC35F1*	12	1.454176	2.23E-12
*CTNNA3*	11	-1.10628	2.5E-07
*MTUS2*	11	-1.06095	3.48E-07
*AGBL1*	10	1.507887	3.07E-07
*ARHGEF4*	10	1.148687	1.39E-11
*TOX*	10	1.469056	8.61E-05

**Fig. 3:**
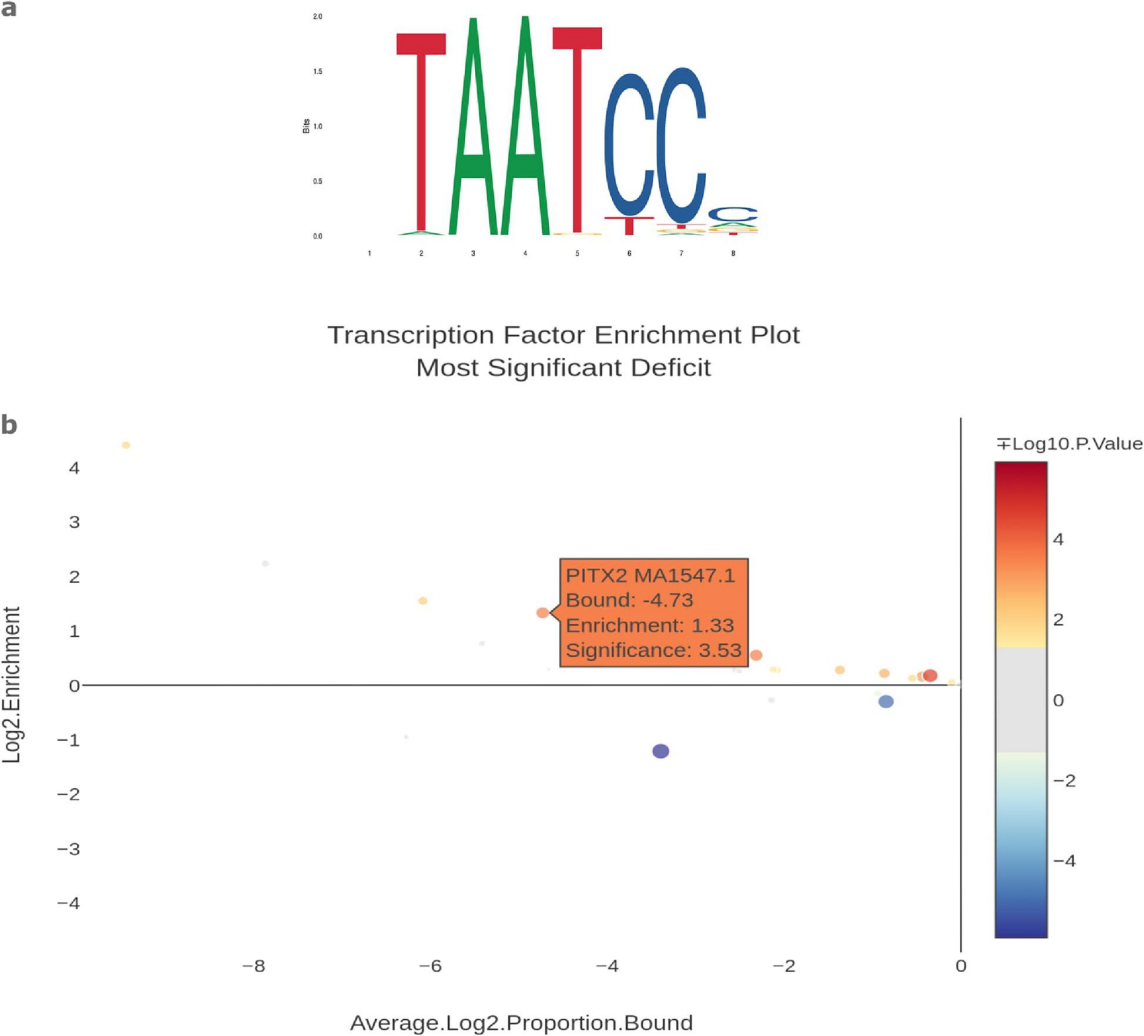
A graphical representation of the position frequency matrix of the *PITX2* transcription factor (**a**). The transcription factor binding site enrichment analysis of differentially expressed transcription factors found in *M.complexus*, focussing on *PITX2*. Genes from eypression genome wide association studies with genome wide significant associations were used here

Enrichment analysis highlighted 5 genes with transcription factor binding sites were significantly enriched in the DEGs of the *M.complexus* of *Na/Na* embryos. These were identified as *PITX2*, *KLF, HES1*, *POU3F1,* and *EN2.* While *PITX2, KLF and HES1* were all observed to have an increased number of transcription factor binding sites, *POU3F1* and *EN2* both had fewer binding sites. *PITX2* showed the most significant enrichment in the measured samples (Fig. 3).

## Discussion

In this study, we compared the liver and *M. complexus* of 6 *Na/Na* and 6 *Wt* chicken embryos in a transcriptomics approach to identify differentially expressed genes. To our knowledge, this is the first transcriptional study of the *M. complexus* of *Na/Na* chickens, building on previous studies that hypothesised a difference in the muscle development of the *M. complexus* plays a role in the hatchability of *Na/Na* chicken embryos [17, 46]. Further raising the question of whether the naked neck locus exerts a direct pleiotropic effect on feather cover and hatchability via developmental impairment of the hatching muscle [47, 48].

We observed DE genes linked to focal adhesion and muscle contraction (*MYLK4, PAK1, PAK5,* and *MY10*) [49]. These are essential for normal embryonic development, for linear growth and for the actual hatching [50]. In addition, cell adhesion molecules (*CLDN1, CLDN19, CLDN25*) [51] and collagen trimers* (COL4A3, COL9A1, COL9A3, COL4A5, COL6A6, COL4A4*) [52, 53], which play pivotal roles in cell structure formation in hatching muscle, were also observed to be down regulated. These genes can affect tight junction-specific obliteration of the intercellular space, through calcium-independent cell-adhesion activity and the retardation of normal embryonic development of the skeleton [51–54]. A significant interaction network involved in contraction regulation was observed, (*TNNI1, TNNT2, MYl3, MYL10, PLN, ATP2A2*). Furthermore, the down regulation of other related genes was observed. Specifically, genes that modulate muscle contractility (*PLN*) [55], myosin regulation (*MYL3, MYL10, MYLK4, MYO7L2, MYH7)* [56, 57], and skeletal muscle contraction through troponin regulation, (*TNNT2, TNNI1*) [55, 58] were all down regulated in *Na/Na* embryos.

The ability of *Na/Na* chickens to minimise stress levels and further impacts of higher ambient temperatures such as a reduction in growth and meat production, or feed efficiency has been previously documented [6, 17, 59]. But the main focus of these studies was the effect a reduced plumage had on their ability to tolerate higher temperatures. A number of studies have focussed on the effect of the *Na/Na* gene on laying performance [59–62], but there has only been a limited use of transcriptional approaches to elucidate the effect of the *Na/Na* gene [29]. *Na/Na* is a cis-regulatory mutation caused by a large insertion (~ 180bp) downstream of the *GDF7* gene [30]. Importantly, here we also observed significantly elevated expression levels of *GDF7* in the *M. complexus* of *Na/Na* embryos (Fig. 1a). *GDF7* is reported to have a tissue specific effect, where elevation of its expression levels in avian skin can suppress the development and distribution of feathers through the sensitizing action of RA [29, 30]. However, the significant elevation of *GDF7* in the *M.complexus* shows that the putative mutation of the *GDF7* gene has a pleiotropic effect on the development of *Na/Na* embryos, likely through the subsequent increase in RA action that affects both skeletal and muscular development, and on the activation of the *M.complexus* directly.

The presence of gene clusters linked to AA-biosynthesis (*PSPH, ASNS, PSAT1,CBSL*) [63], suggest not only slower/ongoing development in these *Na/Na* animals, but also reasons for their potential tolerance and hatchability in hotter environments, where excretion and metabolism of minerals and AAs play a large role [64–66]. This does not however discount changes that affect plumage, as genes linked to diverse feather formation were also differentially expressed in the *Na/Na* (*MMRN1, SOX10*) [67].

To further elucidate some of the potential effects of RA we analysed the RAREs linked to the *M. complexus*. Here we found 240 genes with RAREs in close proximity, with 15 genes being found close to 10 or more RAREs. Two of these genes, *LSAMP* and *MDGA2*, were linked to the immunoglobulin superfamily, and cell adhesion. Four of the up regulated DE genes in *Na/Na* chickens were also linked to embryonic growth (*SOX10, OC3, FSTL5, NLGN3*). These transcriptional changes indicate that there is an observable difference in the development of *Na/Na* and *Wt* embryos.

*Na/Na* chickens also present with a depressed hatchability at so called ‘normal’ temperatures (21-25°C) [68]. Once under increased temperature stresses (30°C) however, *Na/Na* chickens outperform *Wt* animals for hatchability [69]. It is possible that a combination of the DE genes discovered here underlie this, but further study is needed to elucidate. Additionally, the strength of the eggshell is stronger in *Na/Na* chickens [61] . Up regulation in DE genes linked to amino acid biosynthesis pathways could play a role in increasing the thickness and hardness of the eggshells [70]. The up regulation of genes related to calcium transport and binding such as *ATP2A2*, could also play a role, as specific alleles of similar genes have been linked to egg quality measurements [71]. In fact, when environmental temperatures are increased, eggshell thickness and strength is enhanced for *Na/Na* compared to *Wt* chickens [61]. Essential AA are required for optimal growth in broiler chickens, with recommended crude protein levels in feed between 21 and 23% [72]. The up regulation of genes linked to AA biosynthesis (*SLC43A2, SLC38A4, LACAAT2L, GABRP, SMOX*), and serine/threonine protein kinases (*MYLK3, TNNI3K, PCSK1, PCSK4, MAP3K8*), could be linked to *Na/Na* chickens having an optimised ability to metabolise the crude protein under increased temperature environments.

A closer look at the enrichment of transcription factor binding sites shows *PITX2* binding sites as being significantly enriched in both the liver and the *M. complexus*. As *PITX2* is linked to skeletal and muscle development, increases in binding site numbers suggests that unlike the *Wt*, the *Na/Na M. complexus* is not yet fully matured. A further observation was the reduction of enrichment undergone by *HES1* with significantly fewer observed binding sites than expected. This may indicate an innate resistance to heat shock, something that is known to be present in the *Na/Na* chickens [5, 6, 13]. Previous studies have focussed on its lack of neck feathering as the reasoning for this increased heat tolerance but these observed differences may indicate the role of transcriptional factors on this increase, but further work is needed.

## Conclusion

This work represents the first transcriptional study of the *M. complexus* of *Na/Na* chickens. Using a sample of 12 chickens, 6 *Na/Na* and 6 *Wt*, it was possible to observe significant changes in the gene expression. We have shown changes in the regulation of a number of pathways involved in normal embryonic development, as well as amino acid biosynthesis, with likely negative effects on the development of the *M. complexus* in *Na/Na* animals. *GDF7* is already known to suppress the development and distribution of feathers through the sensitizing action of retinoic acid. As such, it is probable that it also has a pleiotropic effect, not just in the reduced feather patterning, but also in the reduced hatchability observed in these embryos. We have also shown a number of target genes, upregulated in *Na/Na* chickens, that are highly likely to play a role in the ability of *Na/Na* chickens to tolerate higher ambient temperatures. This work lays the foundation for further studies into the abilities of *Na/Na* chickens to tolerate the climate related changes that play a clear and present danger to the future of broiler chickens.

## Supplementary Information

Supplementary Material 1.

Supplementary Material 2.

Supplementary Material 3.

Supplementary Material 4.

Supplementary Material 5.

Supplementary Material 6.

## Data Availability

The raw sequencing data supporting the conclusions of this article is available in the NCBI Sequence Read Archive, under BioProject Accession ID: PRJNA1148946 and can be found at: https://www.ncbi.nlm.nih.gov/bioproject/PRJNA1148946.

## Abbreviations

*M.complexus*: *Musculus complexus*
*M. pectoralis superficialis*: *Musculus. pectoralis superficialis*
Wt: Wildtype
*PITX2*: Paired like homeodomain 2
*Na*: Naked Neck
AA: Amino acid
DE: Differentially expressed
RAREs: Retinoic acid response elements
BMP: Bone morphogenetic protein
GO: Gene ontology
PFM: Position frequency matrix
ncRNA: Non-coding ribonucleic acid
KEGG: Kyoto encyclopedia of genes and genomes
