# Supplementary material for: Transcriptional profiling of the M. complexus in naked neck chickens suggest a direct pleiotropic effect of GDF7 on feathering and reduced hatchability
Source: BMC Genomics. 2024 Nov 15;25:1092. doi: 10.1186/s12864-024-10965-0 (PMC11566174; doi:10.1186/s12864-024-10965-0)
Supplement: Supplementary file 2 — Supplementary Material 2. [file 12864_2024_10965_MOESM2_ESM.pdf]

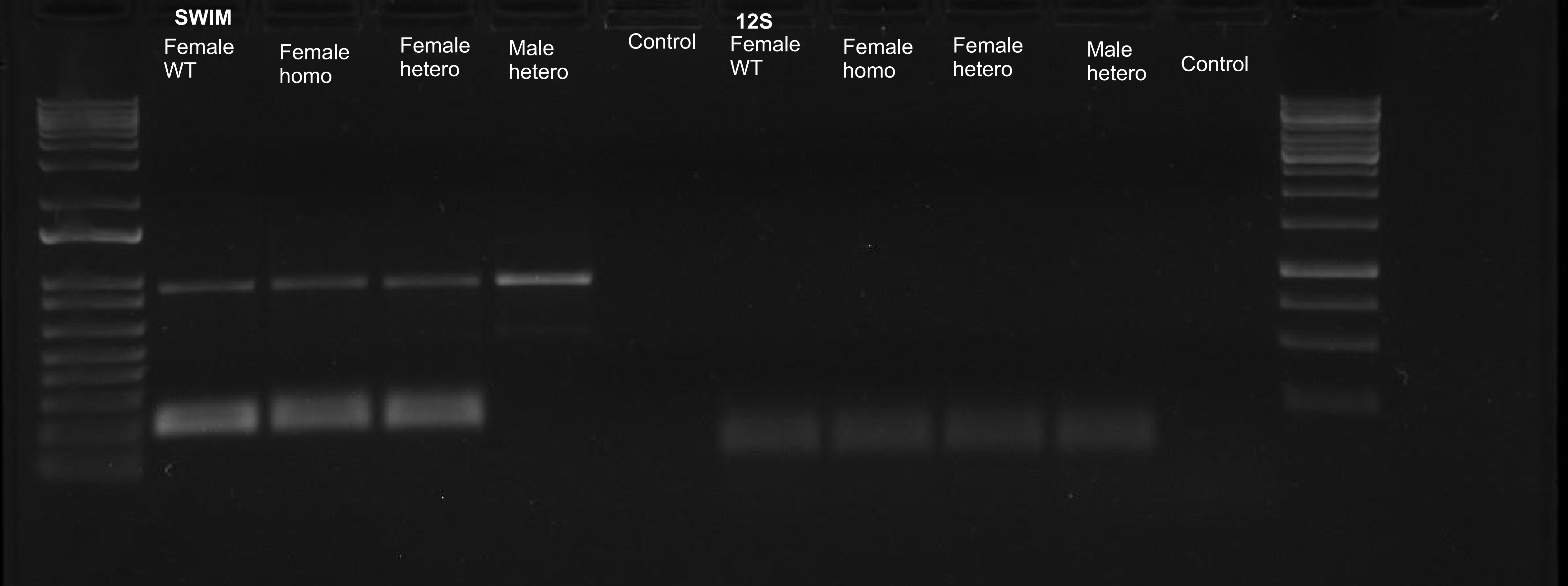

Supplementary file S2. Gel electrophoresis of Multiplex PCR, indicating sex determination using SWIM and 12S primer pairs , 1kb DNA ladder.
